# Complete Genome Sequence of *Leptospira alstonii* Serovar Room22 Strain GWTS #1

**DOI:** 10.1128/genomeA.01230-16

**Published:** 2016-11-10

**Authors:** Jarlath E. Nally, Darrell O. Bayles, Daniel Hurley, Séamus Fanning, Barry J. McMahon, Zbigniew Arent

**Affiliations:** a Infectious Bacterial Diseases Research Unit, National Animal Disease Center, Agricultural Research Service, United States Department of Agriculture, Ames, Iowa, USA; b UCD-Centre for Food Safety, School of Public Health, Physiotherapy & Sports Science, University College Dublin, Belfield, Dublin, Ireland; c School of Agriculture & Food Science, University College Dublin, Belfield, Dublin, Ireland; d University Centre of Veterinary Medicine JU-UAK, University of Agriculture, Krakow, Poland

## Abstract

We report here the complete genome sequence of *Leptospira alstonii* serovar Room22 strain GWTS #1. This is the first isolate of *L. alstonii* to be cultured from a mammal and in western Europe, and it represents a new serovar of pathogenic leptospires.

## GENOME ANNOUNCEMENT

Leptospirosis is a global zoonotic disease caused by pathogenic species of *Leptospira* (1). Pathogenic leptospires are divided into 10 species, which include *Leptospira alstonii*, formerly known as *Leptospira* genomospecies 1 (2, 3). Two serovars of *L. alstonii* have been described, serovar Pingchang and serovar Sihuan, both of which were isolated from frogs in China (4). Additionally, four isolates of *L. alstonii* were cultured from soils in Japan and one isolate from an environmental sample in Malaysia (5, 6). More recently, and for the first time, to our knowledge, *L. alstonii* was isolated in western Europe from a mammal, *Crocidura russula*, the greater white-toothed shrew (GWTS), an invasive species first detected in Ireland in 2007 (7). Of 18 trapped GWTS, three were kidney culture positive by dark-field microscopy. Animal experimental procedures were performed in accordance with relevant guidelines and regulations, and as approved by the National Parks and Wildlife Service (NPWS) in Ireland and the Animal Research Ethics Committee at the University College Dublin (AREC-13-24). All three of the isolates had unique restriction enzyme analysis patterns that differed from those of other isolates of *L. alstonii*, or any pathogenic leptospires. In addition, the GWTS leptospire isolates represent a new serovar designated *L. alstonii* serovar Room22. Here, we present the complete genome sequence of *L. alstonii* serovar Room22 GWTS #1.

Four hundred milliliters of liquid pure culture containing *L. alstonii* serovar Room22 GWTS isolate #1 was harvested and genomic DNA purified as previously described (8). Genome sequencing was performed by the Centre for Genomic Research, University of Liverpool. Genomic DNA libraries were generated according to manufacturer’s instructions (PacBio). Three single-molecule real-time (SMRT) cells were used for library sequencing. Sequencing was done using 240-min movie times with P6/C4 chemistry. The SMRT cell data were assembled using the PacBio Hierarchical Genome Assembly Process (HGAP) within the SMRT Analysis (version 2.3.0) software suite. Assembly resulted in two contigs that comprised the two chromosomes of the genome, with an average coverage depth of 167× across the genome. The genome sequence was subjected to one round of error correction and polishing by remapping the sequencing reads and recalling the genome consensus using the Quiver algorithm. The redundant overlapping genome sequence present at the ends of the contigs was used to circularize each chromosome and simultaneously collapse the overlap into nonredundant sequence. The two circularized chromosomes were broken so as to begin at the *dnaA* and *parA* genes for chromosomes I and II, respectively. The genome sequence was further error corrected and polished by a final round of remapping the sequencing reads and recalling the genome consensus using the Quiver algorithm. The fully closed genome was annotated by the NCBI Prokaryotic Genome Annotation Pipeline. The genome comprises two circular chromosomes of 4,105,414 and 486,474 bp containing 3,868 and 474 coding sequences (CDSs) for chromosomes I and II, respectively. The entire genome has an average G+C content of 42% and contains two complete copies of 5S, 16S, and 23S rRNAs, 37 tRNAs, two noncoding RNAs (ncRNAs), and 42 pseudogenes.

### Accession number(s).

The annotated assembly is available in GenBank under the accession numbers CP015217 (chromosome I) and CP015218 (chromosome II).
